# Economic evidence related to the use of community assets in the management of frailty: a systematic review

**DOI:** 10.3389/fpubh.2026.1874340

**Published:** 2026-07-09

**Authors:** Mohammed G. H. Sammour, Helen P. French, Suzanne McDonough, Llinos Haf Spencer, Mary Lynch

**Affiliations:** 1 Faculty of Nursing and Midwifery, Royal College of Surgeons - RCSI, University of Medicine and Health Sciences,, Dublin, Ireland; 2 School of Physiotherapy, RCSI University of Medicine and Health Sciences, Dublin, Ireland; 3 Wales Institute for Health and Social Care (WIHSC), University of South Wales, Glyntaff Campus, Pontypridd, Wales, United Kingdom

**Keywords:** community assets, cost-effectiveness, economic evaluation, frailty, physical activity, social prescription, systematic review

## Abstract

**Background:**

Frailty is characterized by multisystem dysregulations leading to reduced physiological reserve with greater vulnerability to morbidity and mortality. Community asset interventions have emerged as multidimensional approaches to supporting people with frailty that improve quality of life, strengthen social bonds, and promote wellbeing. While community participation is associated with a higher quality of life, robust evidence on the economic effectiveness of these approaches in populations with or at risk of frailty remains scarce.

**Methods:**

A systematic review was conducted following PRISMA 2020 guidelines and registered on PROSPERO. Five databases were searched from January 2000 to August 2025. Two reviewers screened titles, abstracts, and full texts using the Covidence systematic review management software. A narrative synthesis was conducted following Synthesis without Meta-analysis (SWiM) guidelines.

**Results:**

Twelve studies were included in the review. Five studies were cost-effectiveness analyses (CEA), three applied cost-consequence analyses (CCA), three investigated healthcare resource utilization (HRU)/modeling, and one applied cost–benefit analysis (CBA). Results varied according to methodology, outcome measures, and follow-up periods. In the CEA studies, four interventions were reported as dominant (less costly and more effective than usual care). CCA and HRU/modeling evidence reported inconsistent findings but indicated cost reductions through fewer hospitalizations and delayed transitions to long-term care. A modeling study predicted cost neutrality within 5–7 months and substantial 5-year savings. CBA evidence was limited, as only one study was included.

**Conclusion:**

While multi-component community assets offer potential economic benefits over usual care for frailty management, weak evidence and methodological inconsistencies preclude definitive conclusions. High-quality, future research utilizing standardized economic frameworks and, population-specific outcome tools are needed to inform policy and resource allocation decisions.

**Systematic review registration:**

https://www.crd.york.ac.uk/PROSPERO/view/CRD420251018547, CRD420251018547.

## 1 Introduction

Frailty is characterized by multisystem dysregulation, leading to a loss of dynamic homeostasis, reduced physiological reserve, and greater vulnerability to subsequent morbidity and mortality (1). Pre-frailty represents an intermediate, potentially reversible state preceding frailty, characterized by early multidimensional impairments that increase the risk of negative health outcomes (2). The prevalence of frailty varies depending on the model used. For example, the Physical phenotype model (Fried criteria) estimates 12% of people aged over 50 years are frail, while the cumulative deficits model (or accumulation of deficits) suggests a prevalence of 24% (3, 4). These figures carry important implications for global health system demands, as frailty is associated with substantially increased service utilization, including emergency department presentations, and prolonged hospital admissions (5). As population aging accelerates globally, the economic burden of frailty will also escalate (6). This escalation has prompted a structural shift away from traditional, hospital-centered models toward integrated health and social care pathways within the community to optimize resource allocation and ensure long-term system sustainability (7).

Community asset-based interventions have emerged as multidimensional approaches to supporting people with frailty (8). The concept of community assets is intentionally broad and heterogeneous, encompassing both tangible and intangible resources that support health and wellbeing. Community assets may include health-promoting activities such as healthy food or exercise, while also extending to wider social, organizational, and environmental structures that shape community health (9). In practice, these are often accessed via social prescribing, whereby patients are referred to non-clinical services such as walking groups, arts or gardening classes to address their psychosocial needs. Participation in community assets has demonstrated value in attaining clinical outcomes, and associated with a higher quality of life (10, 11), improves mental health, reduces loneliness, and increases engagement in health behaviors (12). Alongside questions of clinical effectiveness, there is a growing need to understand the economic value of community assets.

Economic evaluation offers a structured framework to assess the monetary value of community assets in managing frailty (13). Evidence suggests that the use of community assets may generate £2.14–£8.56 in social and economic value per £1 invested (14), and a minor improvement in Quality Adjusted Life Years (QALYs) (15).

Despite increasing policy interest, significant challenges persist in evaluating community assets, including capturing long-term benefits within short evaluations, and identifying broader societal benefits that extend beyond healthcare cost savings (16). Without clear economic analysis, policymakers lack guidance on the value of investing taxpayer money in community asset programs and when they can yield cost savings. This review can highlight where evidence is lacking and guide future research and inform decision-makers about the potential return on investment of community asset-based interventions in the management of frailty. The overall aims of this systematic review were to identify and synthesize the economic evidence related to the use of community assets in the management of frailty.

### 1.1 Objectives

- To describe and categorize the types of economic evaluations conducted evaluating use of community asset-based interventions (e.g., cost-effectiveness, cost-utility, cost–benefit analyses) in the management of frailty
- To map the types of community assets utilized in frailty management
- To explore which community assets are associated with the greatest economic impact

## 2 Methods

### 2.1 Approach

A systematic review methodology was employed to identify, critically appraise, and synthesize the existing economic evaluation evidence across relevant studies. This approach is particularly appropriate for reviews concerning frailty and community assets, as it provides a transparent, replicable, and methodologically rigorous process for consolidating heterogeneous evidence. The systematic review method facilitates consistent comparison across studies and supports the identification of methodological, contextual, and evidence gaps within the literature. Such standardization enhances the credibility and reproducibility of the synthesis and aligns with internationally recognized best-practice guidelines for evidence reviews.

This systematic review follows Preferred Reporting Items for Systematic Reviews and Meta-Analyses (PRISMA) 2020 guidelines (17). PRISMA was used to ensure transparency and quality of the review. The systematic review was registered on PROSPERO on 29th May 2025 (CRD420251018547), available from https://www.crd.york.ac.uk/PROSPERO/view/CRD420251018547.

This review is framed following the Population, Intervention, Comparator, Outcome, Study Design (PICOS) format. The utilized PICOS framework is presented in Table 1.

**Table 1:** Study eligibility criteria using the PICOS framework.

PICOS items	Inclusion	Exclusion
Population	Frail, pre-frail adults, or the study used inclusion criteria suggestive of frailty	Healthy adults
Intervention	Used community asset-based interventions in the community including physical activity (e.g., exercises), social prescribing programs	Used non-community-based interventions, such as hospital-based or pharmacological, medical, or healthcare professional interventions within a health service
Comparator	Non-community assets-based interventions including usual care	N/A
Outcome	Assessed any economic evidence for using community assets	N/A
Study design	Peer-reviewed publications between January 2000 and August 2025 Experimental and non-experimental study designs including modeling designs	Opinion-based or non-peer-reviewed studies Conference abstracts Publications not written in the English language.

N/A, not applicable.

### 2.2 Data sources and search strategy

Studies were identified from the following databases: PubMed, Embase, Scopus, Web of Science, and CINAHL. These databases were selected for their global coverage of the health and social care literature. The search covered the last 25 years to align with contemporary clinical practice, recent health system structure, and policies. The concept of using community assets as a patient management approach is relatively new (18). The databases were searched using a set of keywords, with database-specific syntax applied to accommodate differences in indexing and search functions. One reviewer (MGHS) conducted a pilot screening of 500 records to assess the performance of the search strategy.

Keywords were developed based on evidence and consultation with the institutional library service and refined based on three main concepts: Frailty, community assets and economic evaluation methods. Frailty and pre-frailty definitions were based on terminology used by authors of the included studies. Community asset terms were derived from the definitions provided by Newstead et al. (19) incorporating all categories listed in their paper as well as broader concepts such as social prescribing. Economic evaluations encompassed commonly used economic methods alongside economic-related terminology to ensure comprehensive coverage of any economic linkage between frailty and community assets.

The search strategies and keywords for the five databases were developed by reviewer MGHS and reviewed by two further authors from the review team (ML and LHS), along with an institutional librarian. The full details on the search strategy are outlined in Supplementary file 1.

### 2.3 Study selection

The screening process was managed using the Covidence Systematic Review Management Software (20). Following the importation of relevant citations, duplicates were removed by the Covidence software. Study selection was conducted in two stages: first, titles and abstracts were screened by all five reviewers (MGHS, ML, LHS, HPF, and SMD). MGHS served as the lead reviewer for all records, while the remaining team members shared the screening tasks as second reviewers. A third reviewer sought to resolve disagreements. Subsequently, in the second stage of screening, the full texts of all potentially eligible studies were retrieved, uploaded to Covidence, and evaluated against the predefined inclusion and exclusion criteria. Reasons for exclusion of full-text studies were documented and are reported in the PRISMA flow diagram.

### 2.4 Quality assessment

The quality of the included studies was assessed using the Joanna Briggs Institute (JBI) Critical Appraisal Tools, specifically the economic evaluation checklist (21). The JBI tool consists of 11 questions that evaluate various aspects of studies, including clarity of research questions, identification of outcomes and economic value, and costs. Each question can be answered Yes, No, Unclear, or Not Applicable. All studies were assessed for quality, and no studies were excluded based on their quality scores. All quality appraisals were conducted by two reviewers (MGHS and LHS) and presented in Supplementary file 4.

### 2.5 Data extraction

A standardized data extraction instrument within Covidence was developed to extract relevant information based on review objectives. The final data extraction form consisted of 47 items organized into five key domains: (1) study identification, (2) population and clinical context, (3) intervention and comparator characteristics, (4) economic evaluation methodology, and (5) clinical outcomes and economic results. Before the full extraction process, pilot data extraction was conducted by all reviewers on a sample of three studies to standardize the data extracted, identify additional information required, and refine the phrasing of questions.

Data from each eligible study were extracted by two reviewers independently (MGHS, ML, SM, LHS, HPF) using the predefined data extraction form and following consensus on data extracted agreed among the team. The detailed data extraction table can be found in Supplementary file 5.

### 2.6 Data analysis

Data extracted via Covidence were exported and downloaded into Microsoft Excel for systematic analysis. Due to the variation in study methods, results, frailty measures, intervention types, economic evaluations, and outcomes in the extracted data, meta-analysis was not feasible. Therefore, a thematic narrative synthesis was conducted in accordance with SWiM in systematic reviews (22).

The primary economic outcomes, such as Incremental Cost Effectiveness Ratios (ICERs), total costs, and net benefits, were summarized and described across all the studies. Studies reported costs in multiple currencies (GBP, Euros, AUD, CAD) across price years ranging from 2004 to 2020. To enable consistent comparison of economic outcomes, all costs were standardized to 2026 values using a two-step approach. First, costs were inflated to 2026 price levels using country-specific consumer price indices (CPI). Second, the inflated costs were converted into Euros (€), GBP (£), and USD ($) using purchasing power parities (PPP).

### 2.7 Ethical statement

Ethical approval was not required as this review involved the analysis of publicly available information and did not include any interaction with participants.

## 3 Results

### 3.1 Overview of the included studies

**Figure 1:**
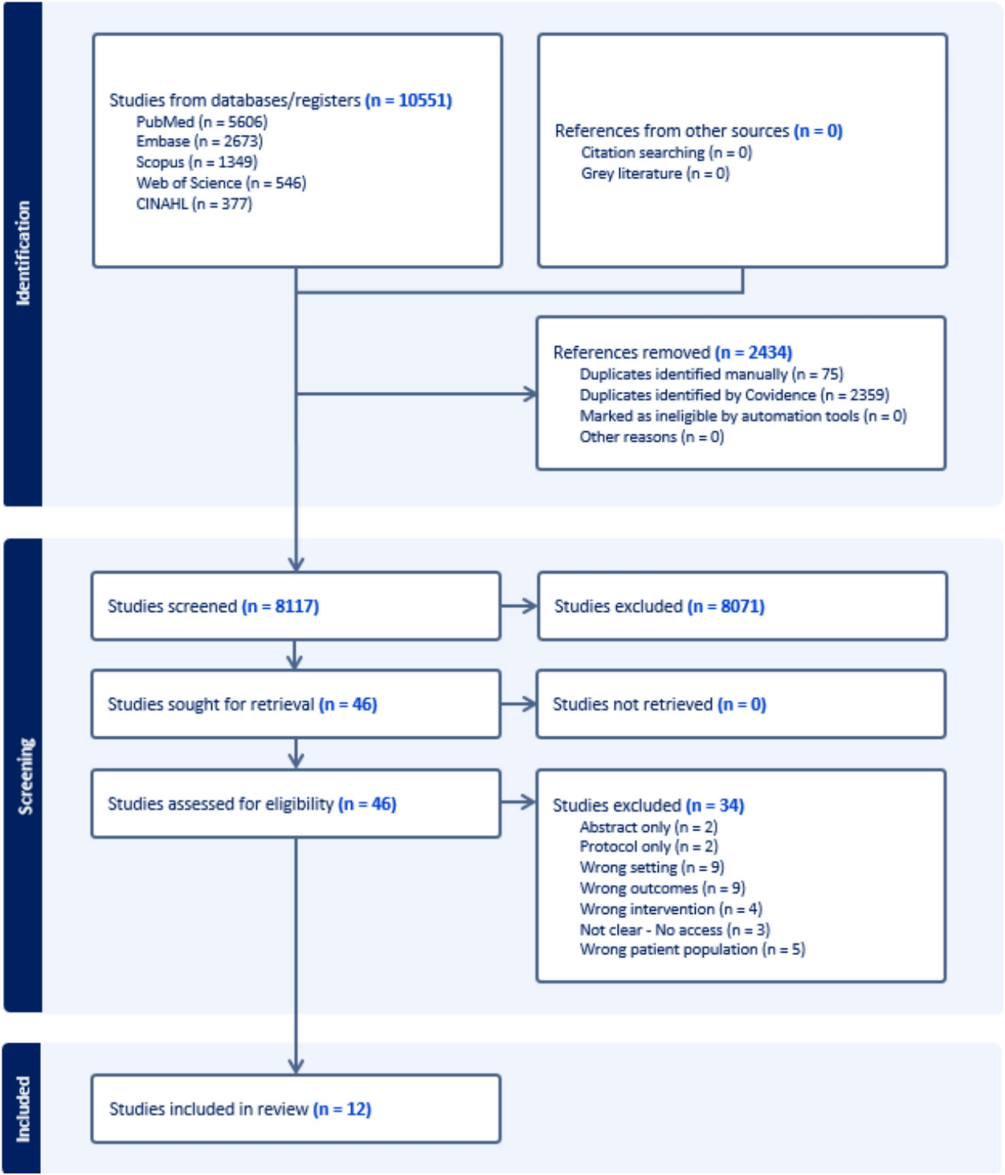
Flow diagram of study selection.

An overview of the included studies is shown in the PRISMA diagram (see Figure 1).

A total of 10,551 studies were identified and imported into Covidence software. Following automated removal of duplicates, 8,117 studies were screened by title and abstract. Of these, 46 studies were retrieved for full-text screening.

Twelve studies that met the inclusion criteria were included in the final synthesis, and 34 were excluded. Eleven of the 12 studies were published between 2017 and 2023, with one published in 2008. The included studies had various designs, with *n* = 7 Randomized Controlled Trial (RCTs), *n* = 2 Quasi-experimental evaluations, *n* = 1 Modeling study, *n* = 1 Cost analysis study, and *n* = 1 Cross-sectional economic analysis.

Studies were predominantly from high-income countries: the UK (*n* = 4), Germany (*n* = 2), multi-nation (Belgium, Czech Republic, UK, France, Germany, Italy, and Spain) (*n* = 1), Canada (*n* = 1), Netherlands (*n* = 1), Spain (*n* = 1), Finland (*n* = 1) and Australia (*n* = 1). Sample size varied across studies, ranging from a small pilot study (*n* = 44) to large-scale registry data (*n* = 190,700). Together, they encompassed more than 220,000 participants across a range of frail or multi-morbid older populations. Follow-up periods also varied: seven studies had long-term follow-up (>12 months) or used modeling for multi-year projections, while six studies reported outcomes in less than a year, which mostly were between 8 and 24 weeks. A summary of the characteristics of included studies (*n* = 12) is in Table 2.

**Table 2:** Characteristics of included studies.

Author and year of publication	Study design	Population	Frailty indicators and definition	Intervention type	Outcome measurement tools	Main economic finding
Adams et al. (29)	RCT	Pre-frail -frailty	The population is visually impaired, able to walk indoors without the help of another person but may use a walking aid, able to walk outdoors but may need the help of another person and/or a walking aid. Given that population may scores 4–6 under clinical frailty score.	Exercise class	Health-related measures (e.g., fear of falling, balance, physical activity, and EQ-5D). In addition to, FES-I, WSAS, FRAT, phone-fit, EQ-5D-5L utilities, ICECAP-O capability score. Frailty was measured as part of study outcome	The average total cost of the intervention per patient across both sites was £310. The capability score in the intervention arm was slightly higher compared to the usual activities arm at baseline. At 12 weeks, capability was slightly higher with both groups having on average 80% capability.
Alhambra-Borrás et al. (30)	Quasi-experimental study	Frail population	Tilburg frailty index	Physical exercise program	Frailty status using Fried Frailty Phenotype, EQ-5D, Physical function measures, including grip strength, walking speed, balance performance, physical activity level, quality of life, GARS, FES-I	The ICER was in the lower-right quadrant. This position of the ICER indicated that the physical activity program was cost-effective in comparison to the usual care alternative.
Dorhout et al. (23)	RCT	Frail population	Fried frailty criteria	Diet and resistance exercise intervention	Short physical performance battery (SPPB), Strength, vitality, mental state, balance, ability to walk, climb the stairs, cycle, reduction in fatigue during daily activities.	An ICER of €2,988 ($3,385)/point increase in SPPB was found. The intervention had an 82.4% probability of being cost-effective at a WTP of €12.000 ($13.559)/point increase in SPPB. Chair-rise test: An ICER of €728/s improvement in chair-rise test was found according to ITT analyses. There was a 99.4% probability of the intervention being cost-effective at a WTP of €20.000/s improvement in chair-rise test.
Dyer et al. (31)	Markov cohort modeling study	Frail/pre-frail population	Cognitively impaired population. Residential dyadic training program for carers and people with dementia. Study arm Frailty Intervention Trial (FIT), includes frail population	Dyadic residential support program	Frailty status mobility	The model predicted that the costs of the programs would break even in approximately 5 months for GTSAH and 7 months for FIT, when compared with standard care, after which the interventions were predicted to save funds
Elston et al. (33)	Cost analysis study	Frail population	Rockwood clinical frailty scale	Resilience-focused coaching and practical support	Before and after costs Wellbeing Star Patient Activation Measure Warwick-Edinburgh Mental Health and Wellbeing Scale Rockwood clinical frailty scale	The total sum of health and social care cost before and after was £387,483 and £749,706, respectively. Thus, there was an overall increase in costs of £362,223 (93.5%). This represented a change in mean cost of £4,212 per user, a statistically significant increase in health and social care.
Jansen et al. (24)	RCT	Not explicitly stated, however, study’s inclusion criteria align with frailty phenotype scale	Frailty Phenotype Scale	fall-prevention exercise interventions (Physical activity program and health promotion activities)	QALYs were calculated based on the EQ-5D-5L.	gLiFE resulted in non-significantly lower QALYs (−0.02, SE = 0.02) and negative ICERs (less effective and more costly), indicating that gLiFE was dominated by LiFE. Conversely, gLiFE participants had a non-significantly higher number of steps/day (+281, SE = 449) and less falls (−0.03, SE = 0.13). From a societal perspective, the ICERs were €3,895 per additional 1,000 steps/day and e39,420 per fall prevented (€1,828 and €18,503 from payer’s perspective).
Lampe et al. (25)	Quasi-experimental study	Pre-frail population	Increased risk for loss of independence measured by the LUCAS functional ability index (LUCAS-FI)	Integrated care approach delivered	Before and after costs Frailty Measures were used Healthcare; outpatient physician consultations, number of hospitalizations, rehab care, pharmaceuticals, number prescriptions of therapeutic devices, non-physician specialist care, nursing home care, patient transportation, number of services	Overall, the mean total health care costs (including intervention costs) were €16,561.80 ± €21,313.94 for participants in the IG and €14,747.77 ± €24,586.94 for participants in the CG, indicating no statistically significant group difference (*p* = 0.051)
Munford et al. (10)	Cross sectional study	Pre-frail population	Older people (>65) with long-term conditions and social care needs indicates pre-frailty population.	Participating in any community assets	HRQoL NHS resource utilization	Participation in community assets is associated with better HRQoL, even after controlling for age, gender, socioeconomic status, and health conditions. When we added in information on the presence of 23 specific health conditions this effect dropped to 0.063 (95% ci 0.048 to 0.077). 0.025 to 0.052;
Rodrigues et al. (26)	RCT	Frail population	Fried frailty index	The Movestrong exercise program and nutrition program	Frailty indicators HRQoL based on EQ-5D-5L Protein and Energy intake Gait speed Chair stand test Dynamic balance	The total cost to administer the program and purchase equipment at all four sites was CAD 377 per participant. The total direct medical cost was CAD 22430, while the total indirect medical cost was CAD 21610.
Rodriguez-Manas et al. (27)	RCT	Frail population	Fried frailty phenotype	Resistance exercise program and structured diabetes and nutritional educational program	Functional performance measured by the SPPB at 12 months. Frailty status EQ-5D-5L	Estimates suggest a mean saving following intervention of €428.02 (2016) per patient per year, with ICER analysis indicating a consistent benefit of the described health care intervention over usual care
Snowsill et al. (28)	RCT	Frail population	SPPB	Group exercise and behavioral maintenance program or three healthy aging education workshops	QALY SPPB EQ-5D-5L	Cost of intervention group = £3,943 The cost of control group = £4,046.
Timonen et al. (32)	RCT	Pre-frail	75 years or older and difficulties in mobility and balance at admission indicate pre-frailty.	Training center or home exercise program	Number of falls	There were no differences between the intervention and control groups in the mean individual healthcare costs, in the social welfare costs, or in the fall-related healthcare costs. The number of training sessions during the 72-week intervention period was 144, and the price for one session was €134.1. For each participant the unit cost of one session was €37.3.

CAD, Canadian dollar; FES-I, Falls Efficacy Scale-International; FIT, Frailty intervention; FRAT, Falls Risk Assessment Tool; GARS, Groningen Activity Restrictions Scale; GTSAH, Residential dyadic training program for carers and people with dementia; HRQoL, Health-related quality of life; ICECAP-O, Investigating Choice Experiments for the Preferences of Older People Capability Measure; ICER, Incremental cost-effectiveness ratio; ITT, Intention-to-treat; NR, Not reported; QALY, Quality-adjusted life year; RCT, Randomized Controlled Trial; SPPB, Short Physical Performance Battery; SD, Standard deviation; WTP, Willingness to pay.

**Table 3:** Monetary valuations for community asset interventions.

Author and year of publication	Economic method	Perspective of analysis	Delivery cost per participant	Benefit/effectiveness	Currency and year	Value in EUR Year of 2026	Value in GBP Year of 2026	Value in USD Year of 2026
Adams et al. (29)	CCA	Healthcare	£310	Compared to baseline, the utility scores at both follow-up periods were improved for both groups but still showed average health-related quality of life scores being poor. The range of utility scores was much larger in the usual activities arm compared to the intervention arm	GBP 2015–2016	€490	£426	$564
Alhambra-Borrás et al. (30)	CEA	Healthcare	€104	Incremental cost (healthcare) -44,832.92 Incremental effects 0.513 ICER (healthcare) - Dominant	Euros 2018	€130	£113	$150
Dorhout et al. (23)	CEA	societal	€1,119	The intervention has an 82.4% probability of being cost-effective at a WTP of €12,000 ($13.559)/point increase in SPPB. The study found an ICER of €2,988 per point increase in SPPB, the intervention has an 82.4% probability of being cost-effective at a WTP of €12,000 per SPPB point increase	Euros 2020	€1,332	£1,159	$1,532
Dyer et al. (31)	Markov cohort modeling study	Healthcare societal	NR	NR	AUD 2018	NR	NR	NR
Elston et al. (33)	CCA	Healthcare societal	£4,212	Increase in mean health costs £2,525. Increase in mean social care costs £1,686. Increase in total cost £4,212 per participant.	GBP 2016–2017	€6,450	£5,612	$7,418
Jansen et al. (24)	CEA	Societal payer	NR	gLiFE had non-significantly higher unadjusted mean costs from societal (+€1,094, SE = €1,184) and payer’s perspective (€513, SE = €798). Regarding the unadjusted effects, gLiFE resulted in non-significantly lower QALYs (−0.02, SE = 0.02) and negative ICER (less effective and more costly)	Euro 2018	NR	NR	NR
Lampe et al. (25)	HRU	Payer	NR	A non-significant mean difference in costs of €1,183 *p* = 0.108 per participant was identified	Euro Year—NR	NR	NR	NR
Munford et al. (10)	CBA	Healthcare societal	NR	Based on a threshold value of £20,000 per QALY, the net benefits of participation in community assets were £763 (95% CI £478 to £1,048) per participant per year, and £1,142 at 30,000 per QALY.	GBP 2014/15	NR	NR	NR
Rodrigues et al. (26)	HRU	Healthcare societal	CAD 377	NR	CAD 2020	€277	£241	$319
Rodriguez-Manas et al. (27)	CEA	Healthcare	NR	Mean savings following the intervention of €428.02	EUR 2016	Mean saving (€554)	Mean saving (£488)	Mean saving ($637)
Snowsill et al. (28)	CEA	Healthcare	£622	Reductions in health and social care usage in delivery costs [£3,943 in the intervention group vs. £4,043 in the control group; difference: £-103 (95% CI £695–£489)]	GBP 2018–2019	€918	£799	$1,056
Timonen et al. (32)	CCA	Healthcare societal	€568	There were no differences between the intervention and control groups in the mean individual healthcare costs: €4,381 vs. €3,539 *p*-value 0.477. In the social costs: €3,336 vs. €4,073 (*P*-value 0.770).	Euro 2004	€888	£773	$1,021

CAD, Canadian Dollar; CEA, Cost-Effectiveness Analyses; CCA, Cost-Consequence Analyses; HRU, Healthcare Resource Utilization; CBA, Cost–Benefit Analysis; CI, Confidence Interval; EUR/€, Euro; GBP/£, Great British Pound; NR, Not reported; QALY, Quality-Adjusted Life Year; SPPB, Short Physical Performance Battery; SE, Standard Error; USD, United States Dollar; WTP, willingness to pay.

A thematic analysis was undertaken to critically synthesize the findings of the studies in line with SWiM guidelines (22). Extracted data relating to intervention type, economic outcomes, measurement tools, and delivery characteristics were synthesized to identify patterns and develop themes that addressed the review objectives. Four overarching themes were identified: (1) types of community asset interventions and their economic outcomes, (2) economic methods and valuation estimates for community asset interventions, (3) measurement tools and outcomes, (4) community asset settings.

### 3.2 Types of community asset interventions and their economic outcomes

Across the included studies (*n* = 12), community asset-based interventions were classified into three categories:

- Multi-component programs (e.g., exercise combined with other programs such as educational or nutritional programs) (*n* = 6) (23–28).
- Physical activity programs (e.g., exercise programs, strength training programs, and fall prevention programs) (*n* = 4) (29–32).
- Health-related programs (e.g., social prescribing and participation in community assets) (*n* = 2) (10, 33).

#### 3.2.1 Multi-component programs

Multi-component programs (23–28) typically combined physical activities with additional support program such as dietary support or education. These studies represented five RCTs (23, 24, 26–28) and one quasi-experimental study (25). Several programs (23, 26–28) were cost-saving compared to usual care. For example, the Pro-Muscle in Practice intervention improved muscle mass and strength while also reporting lower direct health care costs compared to the control group (€1,336 and €1,697, respectively). Although no differences in QALYs were observed in the study, the Short Physical Performance Battery (SPPB) showed an 82.4% probability of being cost-effective (23). Similarly, a group physical activity and health promotion program delivered in the community found improvement in physical activity, and a reduction in falls (24). The evidence reported that the group-based multimodal exercise intervention showed a consistent benefit of the health care intervention over usual care and was dominant (less costly, more effective), with consistent findings across sensitivity analyses (27). Likewise, Snowsill et al. (28) evaluated a 12-month community group physical activity and behavior-maintenance program (REACT) for older adults at risk of mobility decline. Similarly, Lampe et al. (25), who assessed a multi-component community intervention using statutory health-insurance claims and healthcare utilization cost, found no significant impact on total health care costs (€1,183 *p* = 0.108).

The physical activity component within the multi-component programs appeared to be the main contributor to effectiveness (23, 24, 26–28), while non-physical activity components were less robustly supported, as they either demonstrated a small effect or were not statistically significant. There was evidence of cost-effectiveness or favorable economic profiles, particularly when reductions in falls or frailty were considered. However, other findings reported improvements in physical or psychological outcomes without demonstrating clear financial savings or cost-effectiveness (24, 25).

#### 3.2.2 Physical activity programs

Physical activity programs (29–32), which focused solely on physical activity in community settings were represented in *n* = 3 RCTs (29, 30, 32) and *n* = 1 modeling study (31). Economic findings from these programs were less consistent as they reported cost-efficiency, particularly by reducing falls, while others reported improvement in physical outcomes but not financial savings, which was often linked to either limited sample size or duration of the study. For example, Alhambra-Borrás et al. (30) reported on a group-based physical exercise program. The effectiveness analyses showed a significant reduction in the risk of falling (−45.5%; *p* = 0.000) and frailty (−31%; *p* = 0.000) after the intervention. Similarly, Timonen et al. (32) reported that the exercise intervention was effective in improving physical fitness and mood but did not result in any financial savings. Conversely, Adams et al. (29) evaluated an adapted Falls Management Exercise program, which comprised a weekly 1-h group exercise class over 12 weeks held in community venues, and found no clear effect on the clinical outcome.

#### 3.2.3 Health-related programs

Health-related programs (10, 33) focused on broader community participation or social prescribing and were evaluated in two studies: a before-and-after intervention study (33) and a cross-sectional study (10). Elston et al. (33) evaluated the impact of “holistic” link-workers on service users’ wellbeing, frailty, use of health and social care services, and associated costs. The improvement in wellbeing was associated with a statistically significant cost increase (£4,212 per user). Munford et al. (10), who examined the association between participation in community assets and health-related quality of life (HRQoL) and healthcare usage, found that the HRQoL scores were 0.081 (95% CI 0.064–0.098) points higher than those of non-participants. However, the reductions in healthcare usage and costs associated with community asset participation were not statistically significant.

#### 3.2.4 Overall synthesis

Overall, community asset programs, particularly those incorporating physical activity, generally demonstrate promising clinical outcomes and potential economic value. The studies differed in design (RCTs, quasi-experimental, small pilot studies, and modeled evaluations), and economic approach (CEA, CCA, CBA). Multi-component programs were the most reported and showed cost-effectiveness (23–28). However, the evidence for health-related programs (10, 33) was limited as only two studies were identified, which reported inconsistent economic outcomes. Despite methodological heterogeneity and outcome variations, physical interventions in community settings to mitigate frailty showed promise for reducing health and social care costs.

### 3.3 Economic methods and valuation estimate for community asset interventions

Monetary valuations for community asset interventions are presented in Table 3.

The evidence presented in Table 3 outlines the economic evaluation characteristics of the included studies, highlighting heterogeneity in methods, perspectives, and benefits. A range of economic evaluation methods were applied across the 12 included studies. In total, there were *n* = 5 CEA (23, 24, 27, 28, 30), *n* = 3 CCA (29, 32, 33), *n* = 3 HRU/Modeling (25, 26, 31), and *n* = 1 CBA (10). Studies were conducted from healthcare, societal, and payer perspectives, sometimes within the same study. Table 2 shows evaluations that varied in their ability to provide direct valuation estimates in monetary terms and outcomes measured included SPPB scores, number of falls prevented, QALYs, frailty transitions (from frail to pre-frail), and healthcare utilization.

Intervention delivery costs varied significantly, driven by intervention intensity, duration, logistical requirements, and workforce-related costs. Reported cost per participant varied significantly from as low as €104 (or €130, 2026 price) for a group-based multi-component physical exercise program delivered twice weekly over 5 months (30), to £4,212 (or €6,450–2026 price) for resilience-focused coaching and practical support program (33), with several studies not reporting delivery cost at all (10, 24, 25, 27, 31).

Across studies, personnel costs were the primary cost driver. Trained professionals represented the largest cost component in all studies including trained professionals, physiotherapists (28–30), study coordinators (25) and dietitians (23). Venue costs were the second highest cost driver.

Evidence from CCA and HRU studies (25, 26, 29, 32, 33) reported inconsistent findings as shown in Table 3. One study reported a non-significant mean cost difference of €1,183 per participant (*p* = 0.108) (25), while another identified increases in mean health costs (£2,525), social care costs (£1,686), and total costs (£4,212) per participant (33). Conversely, another study demonstrated no statistically significant differences between intervention and control groups (32). Interpretation of these findings should be cautious, as these five studies (25, 26, 29, 32, 33) varied in methodological quality and did not consistently fulfill a high number of JBI appraisal criteria. JBI checklist fulfillment ranged from 2 to 8 items (Supplementary file 4), suggesting uncertainty regarding the robustness of the available evidence.

Evidence on net-benefit and social value was limited. One study used a social net-benefit framework and estimated the net benefit of £763–£1,142 per participant associated with community asset participation (10). Research examining direct health and social welfare cost supporting community programs for older individuals was financially beneficial, attaining cost neutrality within a 5-7-month period and overall net savings over 5 years (31).

Table 4 summarizes the economic findings from the included studies. Results varied according to outcome measure, follow-up periods, and WTP threshold. Five studies reported ICERs that can be compared against established cost thresholds (23, 24, 27, 28, 30). However, these ICERs were derived using heterogeneous outcomes (e.g., SPPB, and chair-rise performance), limiting direct comparison across studies. The evidence reported interventions dominant (i.e., more effective and less costly than usual care). For example, a study reported cost-effectiveness with a WTP of €12,000 per point increase in SPPB, and this percentage rises to 99.4% at €20,000 for chair-rise test (23). Notably, all five studies score equal or greater than 9 out of 11 in JBI quality assessment, suggesting relatively strong methodological quality.

**Table 4:** Cost-effectiveness and economic outcomes.

Author and year of publication	Outcome measure	ICER	Cost threshold	Probability cost-effective	Follow-up	Key economic findings
Alhambra-Borrás et al. (30)	Frailty improvement	Dominant	NR	NR	5 and 9 months	Hospitalization −50%, GP visits −33%
Dorhout et al. (23)	SPPB improvement	€2,988	€12,000	82.40%	24 weeks	Lower physiotherapy use
Dorhout et al. (23)	Chair-rise test	€728	€20,000	99.40%		
Jansen et al. (24)	Fall prevention (payer)	€18,503	Acceptability curves	Non-significant	NR	Costs higher, effects lower
Jansen et al. (24)	Fall prevention (societal)	€39,420	NR	NR	NR	NR
Rodriguez-Manas et al. (27)	SPPB improvement	Dominant	NR	NR	12 months	€428 saving per patient
Snowsill et al. (28)	QALY gained	£17,000	£20,000–30,000	High	–	Reduced health/social care use

GP, General Practitioner; NR, Not reported; QALY, Quality-Adjusted Life Year; SPPB, Short Physical Performance Battery.

Similarly, two studies (27, 30) suggested that their physical activity program may provide economic benefits alongside improvements in physical function, including mean savings of €428.02 per patient per year (27), and reduction in hospitalization costs (30). Jansen et al. (24) adopted more than one analysis perspective and demonstrated a clear difference between ICER based on the perspective of €39,420 vs. €18,503 per fall prevented for societal and payer, respectively.

Overall, the available findings suggest potentially favorable and dominant cost-effectiveness associated with some community asset interventions. This was demonstrated by reductions in healthcare utilization and potential long-term cost savings. However, the diversity in study design, economic evaluation methods, perspective (healthcare, payer, societal), and time horizon (short within-trial follow-up versus modeled long-term) and outcome measures restrict definitive conclusions regarding economic outcomes.

### 3.4 Measurement tools and outcomes

Various measurement tools were used across the 12 studies to assess quality of life, frailty status, physical activity, and function. The most frequently applied tool was EQ-5D-5L, which was the predominant HRQoL measure used in eight of the included studies (10, 23, 24, 26–30). In addition to Quality of Life (QoL), several studies assessed frailty status, physical activity, and function, and mobility using multiple physical performance and functional measurements such as grip strength, gait speed, and functional mobility tests (23, 25–27, 29–33). Further details are provided in Supplementary file 5.

All eight included studies (10, 23, 24, 26–30) which used EQ-5D-5 to measure HRQoL reported modest improvements or non-significant changes between the intervention and control group following the use of community assets, despite improvements in physical function. In contrast, Elston et al. (33), using The Warwick-Edinburgh Mental Wellbeing Scale (WEMWBS), reported a meaningful improvement in wellbeing, with 62.9% of participants achieving an increase of five or more points (mean change +20.3%, *p* < 0.001). The remaining studies did not report quality of life outcomes but instead focused on cost or healthcare utilization measures (25, 31, 32).

All studies reported improvements in physical outcomes, however, these outcomes varied across and within the studies in terms of the used measures: ranging from strength measures (grip strength) to functional performance (sit-to-stand, dynamic balance, gait speed) to day-to-day physical activity. Rodrigues et al. reported significant improvements in grip strength, sit-to-stand performance, and dynamic balance, while no significant effects were observed for gait speed or physical activity levels (26).

Four studies used the SPPB to assess changes in physical outcomes (23, 27, 28, 30). Findings indicate that participation in physical exercise programs is associated with improvement in all variables measured through the SPPB (30). In addition, results found after 24 weeks participation in a program there was an incremental effect in physical functioning of 0.3 SPPB points (23) while another study found SPPB scores 0.85 points higher than those in the usual care group (27). Modeling results estimated that an individual aged 75 years undertaking a program could attain an additional 0·317 in life-years spent with a SPPB score of between 8 and 12 (28).

Overall, while community assets interventions demonstrated improvements in physical functioning, these improvements were not consistently reflected in quality-of-life measures, specifically EQ-5D-5L, suggesting potential limitations in the sensitivity of generic QoL tools to understand the benefits of interventions for frail older adults.

### 3.5 Community assets settings

The community assets predominantly included physical activity or exercise programs in community settings. Community settings included leisure centers, training centers, fitness centers, and home-delivered programs. In terms of frequency of usage for the frailty programs, a prominent pattern emerged, of engagement of between 2 and 3 times per week (23, 25–30, 32) across the majority of the included studies, indicating consistent involvement in the structured interventions. However, some studies used a more flexible delivery approach, Dyer et al. (31), had 10-day in-patient carer training before discharge with follow-up over months, while Jansen et al. (24) offered programs without fixed session times or frequency. Interventions were delivered in various formats, including group-based programs, individual sessions, and combined approaches.

Overall, the heterogeneity in the included studies reflects the broad definition of community assets in the literature, indicating flexible and individualized approaches to frailty management. However, these variations also had an influence on how the economic value of the community asset was estimated.

### 3.6 Gaps, limitations, and implications for future research

Several gaps and limitations were identified in this systematic review, driven mainly by heterogeneity in community asset types, types of intervention, economic evaluation methods, and outcome measures, which limited direct comparison between studies:

- In majority of studies, cost-efficiency was not measured, and QALYs were not reported for all studies
- Included studies had small sample sizes leading to more biased, and potentially less robust results
- Studies using EQ-5D-5L generally reported minimal or insignificant improvements in quality of life despite the improvements in physical outcomes, raising concerns about the use of EQ-5D-5L, which is a generic HRQoL questionnaire
- The included studies were primarily conducted in Western high-income countries, resulting in a skewed distribution and limited representation from low- and middle-income regions

In summary, the current evidence regarding community assets and frailty interventions is limited. The small number of included studies highlighted the need for further research using appropriate outcome measurements over a longer follow-up period. Future research should priorities methodological quality, using methods that have been proven to measure the social value of community-based interventions, including social return on investment methods utilizing contingent valuation to support resource allocation. This would assist policymakers to gather robust evidence to make informed decisions regarding the economic value of the use of community assets in managing frailty.

## 4 Discussion

### 4.1 Summary of the main findings

This systematic review synthesized economic evidence from 12 studies examining community assets interventions for frail or pre-frail adults. The findings identified three primary categories of community assets: (1) multi-component programs (*n* = 6), (2) physical activity programs (*n* = 4) and (3) health-related programs (*n* = 2). The evidence was mainly derived from high-income countries, with the UK representing the largest proportion of studies (*n* = 5). The findings demonstrate that community assets interventions may show a favorable economic profile, with several studies reporting cost-efficacy. Nevertheless, the overall economic evidence base remains limited.

The multi-component programs emerged as the most studied intervention type and demonstrated consistent economic benefits, although the magnitude of these benefits varied across studies which could be due to the intervention itself (e.g., dosage), the quality of the study or differences in methodologies. Physical activity programs alone showed promising economic outcomes, while evidence for health-related assets was limited and inconclusive.

Due to the heterogeneity in study designs and economic evaluation methods across the studies, a meta-analysis was not performed; the results are presented narratively. The range of economic evaluation included CEA, CCA, HRU/modeling, and CBA. This diversity mirrors the finding of Wreford et al. that asset-based interventions widely varied (16). The intervention costs varied considerably from €105 to €1,119 per participant. Eight studies lacked long-term costs or provided inadequate details on cost components. This systematic review aligns with previous findings; for example, Pinheiro et al. highlighted that there is a scarcity of reviews investigating the value for money of physical activity interventions (34). It is important to consider the findings of this systematic review alongside variation in methodological quality. Although several studies reported potential favorable outcomes, the certainty of these findings remains limited.

### 4.2 Types of economic evaluations in community asset research

The first objective of this systematic review was to describe and categorize the types of economic evaluations conducted on community asset-based interventions. The use of CEA and CUA in research on frailty and older people in general has faced criticism (35). The CEA was the most common analysis used, representing 46% of the included studies. This aligns with NICE recommendations to use CEA when evaluating health interventions (36). This study type allows the use of ICER to analyze data, enabling comparison with established NICE thresholds (£25,000–£35,000) (37).

Results in this review indicated that while physical outcomes such as SPPB improved, HRQoL as measured by the EQ-5D-5L did not improve (23, 30). This suggests a sensitivity gap in the EQ-5D-5L questionnaire, where it fails to capture the life-changing improvements that frail people care about. These findings are consistent with previous research (38). Health-related programs present an economically complex picture as they are viewed very positively by participants due to their effects on isolation and loneliness (39). Despite this positive finding, in this systematic review, the health-related programs, reported a significant increase in their cost due to more GP appointments, medication reviews, and home visits (33). However, the National Academy for Social Prescribing (NASP) in the UK suggests that for every £1 invested, the social and economic return can range from £2.14 to £8.56 (40). These figures include the social value, which is not usually captured in the overall healthcare budgets.

Frailty interventions may take several years to yield cost-effectiveness (by delaying nursing home admission or prolonging hospitalization) (41), therefore, modeling is essential. Dyer et al. (31) demonstrated that community assets can become cost-neutral within months and cost-beneficial within years. This is an important consideration in community asset intervention studies as it overcomes the relatively short timelines of the studies.

### 4.3 Types of community assets utilized in frailty management

The included studies were dominated by exercise programs delivered in the community, in fitness clubs, or at home. While these programs suggested a benefit for the physical health of the participants, there were non-significant improvements in the EQ-5D-5L quality of life measure. This suggests that exercise mitigates frailty and could reverse pre-frailty status in older adults, and could reduce the progression to frailty, as has been demonstrated widely in previous studies (42–44).

The multi-component intervention led by Rodriguez-Manas et al. (27), which was a RCT with high quality score and delivered to frail and pre-frail participants aged ≥70 years with type 2 diabetes mellitus in community centers, and primary care, is noteworthy as it achieved better health outcomes at a lower total cost.

While 11 studies in this review utilized physical activity programs in community settings, the evidence for the health-related non-exercise programs, such as link worker and social prescribing programs, remains limited. This finding aligns with a previous systematic review (45).

### 4.4 Strengths and limitations

This review has several strengths. The systematic search strategy followed the PRISMA guidelines, which ensures comprehensive study inclusion. This review incorporated a broad range of intervention types and study designs, which provides a real-world perspective on the diversity of frailty interventions. The thematic analysis allowed synthesis of heterogeneous studies while keeping the necessary economic details.

However, the low number of studies (*n* = 12) and study heterogeneity meant that meta-analysis could not be conducted. The geographical predominance of high-income countries could potentially limit the generalizability of the findings and application to low- and middle-income countries. Health system, social care, and community resources vary significantly between high-, middle-, and low-income countries, which may influence the effectiveness and economic value of community assets.

The short-term follow-up periods (less than 12 months in six studies) limits the understanding of the long-term economic impacts of community asset interventions use, which may be substantial given the chronic nature of frailty.

### 4.5 Future research priorities and recommendations

To build a more robust evidence base for the use of community assets use in frailty management, future research should priorities the following key components:

- Considering that not all studies measured cost-efficiency in the use of community assets in the management of frailty, future research should integrate economic evaluation methods such as social return on investment (SROI), which could provide a comprehensive framework for capturing the value for money from a societal perspective
- Given that many studies had small sample sizes, adequately powered studies, with longer follow-up, and societal perspectives are needed to assess the long-term benefits
- As studies that used EQ-5D-5L reported minimal improvements in quality of life despite gains in physical outcomes, multi-dimensional measurements that capture psychosocial benefits of the interventions would help, for example,
  - SPPB: as a sensitive clinical tool for functional impact
  - ICECAP-O or ASCOT: to capture broader capability and social benefits
  - The frailty index: an indicator of multivariate risks
- Further research from low and middle-income countries is needed to inform policy where community assets may be particularly important due to limited resources.
- Limited evidence for health-related programs suggests the need for more economic evaluation of social prescribing and link-worker programs.
- Future research should explore the different implementation strategies, such as group versus individual, professional-led versus peer-led facilitation, and community versus facility-based frailty interventions.

## 5 Conclusion

This systematic review aimed to identify and synthesize the economic evidence related to the use of community assets in the management of frailty. Multi-component programs that combine physical activity with an additional component, such as education or nutrition promotion, were the most commonly evaluated programs. Findings suggest multi-component programs with physical activity as the primary driver of effectiveness, may offer potential economic benefits and could represent promising alternatives to usual care. Given the heterogeneity of study designs, variation in community assets, and inconsistency in economic evaluation, these findings should be considered with caution. Health-related programs and the non-physical components of multi-component community assets require further evaluation to determine which asset-based strategies offer the best return on investment for frailty management.

Although some studies reported favorable economic outcomes, significant gaps in the evidence were identified, including limited use of comprehensive economic evaluation methods, small sample sizes, short follow-up periods, and measurement tools that may not fully capture the benefits related to frail populations.

In summary, while community assets show potential as effective strategies for frailty management, the current evidence base is not sufficiently robust to draw definitive conclusions regarding cost-effectiveness. Further high-quality research is required. Future studies should use standardized economic methodologies and utilize sensitive and multidimensional outcome measures to better inform policy and resource allocation.

## References

[1] Ageing WCCoH. Report of Consortium Meeting in Geneva, Switzerland: Ageing and Health team, WHO (2017).

[2] Sezgin D Liew A O'Donovan MR O'Caoimh R. Pre-frailty as a multi-dimensional construct: a systematic review of definitions in the scientific literature. Geriatr Nurs. (2020) 41:139–46. doi: 10.1016/j.gerinurse.2019.08.004, 31466806

[3] Xue QL. The frailty syndrome: definition and natural history. Clin Geriatr Med. (2011) 27:1–15. doi: 10.1016/j.cger.2010.08.009, 21093718 PMC3028599

[4] O'Caoimh R Sezgin D O'Donovan MR Molloy DW Clegg A Rockwood K . Prevalence of frailty in 62 countries across the world: a systematic review and meta-analysis of population-level studies. Age Ageing. (2021) 50:96–104. doi: 10.1093/ageing/afaa219, 33068107

[5] Nari F Park E-C Nam C-M Jang S-I. Impact of frailty on mortality and healthcare costs and utilization among older adults in South Korea. Sci Rep. (2023) 13:21203. doi: 10.1038/s41598-023-48403-y, 38040759 PMC10692079

[6] Chi J. Chen F. Zhang J. Niu X. Tao H. Ruan H. Impacts of Frailty on Health Care Costs Among Community-Dwelling Older Adults: A Meta-Analysis of Cohort Studies. Lanzhou, China: Elsevier (1872). 10.1016/j.archger.2021.104344 33516075

[7] Giusti M De Remigis S Greco S Profeta VF Bonaccorsi G Zanobini P . Integration of resources in social and healthcare services for ensuring the continuity of care to frail individuals aged 65 or over: an Italian experience. Front Public Health. (2025) 13:1562564. doi: 10.3389/fpubh.2025.1562564, 40469610 PMC12135681

[8] Chen IA-O Chen YA-O Phanumartwiwath AA-O. Community-Based Care Interventions for Frail Older Adults: A Scoping Review of Multidimensional Strategies Across Pandemic Eras. Bangkok, Thailand: Wiley (2026). 10.1111/phn.70071 41540891

[9] Ravaghi H Guisset AL Elfeky S Nasir N Khani S Ahmadnezhad E . A scoping review of community health needs and assets assessment: concepts, rationale, tools and uses. BMC Health Serv Res. (2023) 23:44. doi: 10.1186/s12913-022-08983-3, 36650529 PMC9847055

[10] Munford LA Sidaway M Blakemore A Sutton M Bower P. Associations of participation in community assets with health-related quality of life and healthcare usage: a cross-sectional study of older people in the community. BMJ Open. (2017) 7:e012374. doi: 10.1136/bmjopen-2016-012374, 28183807 PMC5306503

[11] Bower PRD Sutton M Sutton M Lovell K Blakemore A Hann M . Improving Care for Older People with Long-Term Conditions and Social Care Needs in Salford: The CLASSIC Mixed-Methods Study, Including RCT. Salford, the UK: NIHR Journals Library (2018). 30183219

[12] Sadio R Henriques A Nogueira P Costa A. Social prescription for the elderly: a community-based scoping review. Prim Health Care Res Dev. (2024) 25:e46. doi: 10.1017/S1463423624000410, 39417591 PMC11569851

[13] Haji Ali Afzali H Karnon J Theou O Beilby J Cesari M Visvanathan R. Structuring a conceptual model for cost-effectiveness analysis of frailty interventions. PLoS One. (2019) 14:e0222049. doi: 10.1371/journal.pone.0222049, 31509563 PMC6738928

[14] Polley M. Seers H. Toye O. Henkin T. Waterson H. Bertotti M. . Building the Economic Evidence Case for Social Prescribing London: University of East London (2023).

[15] Moffatt SWJ Pollard TM Gibson K Wildman JM O'Brien N Griffith B . Impact of a Social Prescribing Intervention in North East England on Adults with Type 2 Diabetes: The SPRING_NE Multimethod Study. Southampton, UK: National Institute for Health and Care Research (2023). 10.3310/AQXC8219 37254700

[16] Wreford A Birt L Whitty JA Hanson S Conquer S Wagner AP. Cost and economic evidence for asset-based approaches to health improvement and their evaluation methods: a systematic review. BMC Public Health. (2024) 24:814. doi: 10.1186/s12889-024-18231-4, 38491442 PMC10941621

[17] Page MJ McKenzie JE Bossuyt PM Boutron I Hoffmann TC Mulrow CD . The PRISMA 2020 statement: an updated guideline for reporting systematic reviews. BMJ. (2020) 372:n71. doi: 10.1136/bmj.n71 PMC8005924 33782057

[18] Council BHC. Community Assets Brighton & Hove. UK: Brighton & Hove City Council (2013).

[19] Newstead S WC Jenkins B Lavans A Jesurasa A A Glossary of Terms for Social Prescribing in Wales. (2023). Available online at: https://splossary.wales/wp-content/uploads/2023/12/A-GOT-for-SP-in-Wales_English-December-2023_Final.pdf (Accessed June, 30, 2026). 10.5334/ijic.8591 PMC11225557 38974206

[20] Covidence. Covidence. Available online at: https://www.covidence.org/ (Accessed June, 30, 2026).

[21] Hilton M. JBI Critical Appraisal Checklist for Systematic Reviews and Research Syntheses. Canada: Journal of the Canadian Health Libraries Association (2026).

[22] Campbell M McKenzie JE Sowden A Katikireddi SV Brennan SE Ellis S . Synthesis without meta-analysis (SWiM) in systematic reviews: reporting guideline. BMJ. (2020) 368:l6890. doi: 10.1136/bmj.l6890, 31948937 PMC7190266

[23] Dorhout BG Haveman-Nies A van Dongen EJI Wezenbeek NLW Doets EL Bulten A . Cost-effectiveness of a diet and resistance exercise intervention in community-dwelling older adults: ProMuscle in practice. J Am Med Dir Assoc. (2021) 22:792–802.e2. doi: 10.1016/j.jamda.2020.12.036, 33548182

[24] Jansen C-P Gottschalk S Nerz C Labudek S Kramer-Gmeiner F Klenk J . Comparison of falls and cost-effectiveness of the group versus individually delivered lifestyle-integrated functional exercise (LiFE) program: final results from the LiFE-is-LiFE non-inferiority trial. Age Ageing. (2023) 52:afac331. doi: 10.1093/ageing/afac331, 36702515

[25] Lampe D Hasemann L Nebling T Thiem U Greiner W. Health economic perspective on a community-based intervention for older people at risk of care dependency - results of a prospective quasi-experimental study. Gerontol Geriatr Med. (2022) 8:23337214221140222. doi: 10.1177/23337214221140222, 36458264 PMC9706052

[26] Rodrigues IB Wagler JB Keller H Thabane L Weston ZJ Straus SE . Encouraging older adults with pre-frailty and frailty to "MoveStrong": an analysis of secondary outcomes for a pilot randomized controlled trial. Health Promot Chronic Dis Prev Can. (2022) 42:238–51. doi: 10.24095/hpcdp.42.6.02, 35766913 PMC9388057

[27] Rodriguez-Manas L Laosa O Vellas B Paolisso G Topinkova E Oliva-Moreno J . Effectiveness of a multimodal intervention in functionally impaired older people with type 2 diabetes mellitus. J Cachexia Sarcopenia Muscle. (2019) 10:721–33. doi: 10.1002/jcsm.12432, 31016897 PMC6711410

[28] Snowsill TM Stathi A Green C Withall J Greaves CJ Thompson JL . Cost-effectiveness of a physical activity and behaviour maintenance programme on functional mobility decline in older adults: an economic evaluation of the REACT (retirement in action) trial. Lancet Public Health. (2022) 7:e327–34. doi: 10.1016/S2468-2667(22)00030-5, 35325628 PMC8967720

[29] Adams N Skelton DA Howel D Bailey C Lampitt R Fouweather T . Feasibility of trial procedures for a randomised controlled trial of a community based group exercise intervention for falls prevention for visually impaired older people: the VIOLET study. BMC Geriatr. (2018) 18:307. doi: 10.1186/s12877-018-0998-6, 30541483 PMC6292024

[30] Alhambra-Borrás T Durá-Ferrandis E Ferrando-García M. Effectiveness and estimation of cost-effectiveness of a group-based multicomponent physical exercise programme on risk of falling and frailty in community-dwelling older adults. Int J Environ Res Public Health. (2019) 16:2086 p. doi: 10.3390/ijerph16122086, 31200434 PMC6617042

[31] Dyer SM Standfield LB Fairhall N Cameron ID Gresham M Brodaty H . Supporting community-dwelling older people with cognitive impairment to stay at home: a modelled cost analysis. Australas J Ageing. (2020) 39:e506–14. doi: 10.1111/ajag.12818, 32609939 PMC7818109

[32] Timonen L Rantanen T Makinen E Timonen TE Tormakangas T Sulkava R. Cost analysis of an exercise program for older women with respect to social welfare and healthcare costs: a pilot study. Scand J Med Sci Sports. (2008) 18:783–9. doi: 10.1111/j.1600-0838.2007.00752.x, 18248543

[33] Elston J Gradinger F Asthana S Lilley-Woolnough C Wroe S Harman H . Does a social prescribing 'holistic' link-worker for older people with complex, multimorbidity improve well-being and frailty and reduce health and social care use and costs? A 12-month before-and-after evaluation. Prim Health Care Res Dev. (2019) 20:e135. doi: 10.1017/S1463423619000598, 31547895 PMC6764188

[34] Pinheiro MB Howard K Oliveira JS Kwok WS Tiedemann A Wang B . Cost-effectiveness of physical activity programs and services for older adults: a scoping review. Age Ageing. (2023) 52:afad023. doi: 10.1093/ageing/afad023, 36934340 PMC10024893

[35] Gottschalk S Konig HH Nejad M Dams J. Measurement properties of the EQ-5D in populations with a mean age of >/= 75 years: a systematic review. Qual Life Res. (2023) 32:307–29. doi: 10.1007/s11136-022-03185-0, 35915354 PMC9911506

[36] NICE Assessing Cost Effectiveness (2012). Available online at: https://www.nice.org.uk/process/pmg6/chapter/assessing-cost-effectiveness (Accessed June 30, 2026).

[37] NICE Changes to NICE’s Cost-Effectiveness Thresholds Confirmed (2025). Available online at: https://www.nice.org.uk/news/articles/changes-to-nice-s-cost-effectiveness-thresholds-confirmed

[38] Nikolova S. Hulme C. West R. Pendleton N. Heaven A. Bower P. Normative Estimates and Agreement Between 2 Measures of Health-Related Quality of Life in Older People With Frailty: Findings From the Community Ageing Research 75+ Cohort Leeds, the UK: Elsevier (2025). 10.1016/j.jval.2020.04.1830 32828218

[39] Liebmann M Pitman A Hsueh YC Bertotti M Pearce E. Do people perceive benefits in the use of social prescribing to address loneliness and/or social isolation? A qualitative meta-synthesis of the literature. BMC Health Serv Res. (2022) 22:1264. doi: 10.1186/s12913-022-08656-1, 36261835 PMC9580419

[40] NASP Building the Economic Case for Social Prescribing: NASP; (2023). Available online at: https://socialprescribingacademy.org.uk/nasps-evidence-reports/building-the-economic-case-for-social-prescribing/ (Accessed June, 30, 2026).

[41] Ji S. Lim J. An TJ Jang G. Baek JY Park K. Long-Term Health and Cost Outcomes of a 24-Week Multicomponent Frailty Intervention in Older Adults. Seoul, Korea: Jamanetwork (2026). 10.1001/jamanetworkopen.2025.43278 PMC12612941 41222933

[42] de Labra C. Guimaraes-Pinheiro C. Maseda A. Lorenzo T. Millán-Calenti JC. Effects of Physical Exercise Interventions in Frail Older Adults: A Systematic Review of Randomized Controlled Trials. A Coruña, Spain: BMC Geriatricss. 10.1186/s12877-015-0155-4 PMC4667405 26626157

[43] Flores-Bello C Correa-Muñoz E Sánchez-Rodríguez MA Mendoza-Núñez VM. Effect of exercise programs on physical performance in community-dwelling older adults with and without frailty: systematic review and meta-analysis. Geriatrics. (2024) 9:8. doi: 10.3390/geriatrics9010008, 38247983 PMC10801556

[44] Lim H. Jani N. D. B. Pang W. T. Lim E. C. W., (2026|). Community-Based Exercises Improve Health Status in Pre-Frail Older Adults: A Systematic Review with Meta-Analysis. Yishun, Singapore: BMC Geriatrics. 10.1186/s12877-024-05150-7 PMC11234756 38987690

[45] Kiely BA-O Croke A. O'Shea M. Boland FA-O O'Shea E. Connolly D. . (2026). Effect of Social Prescribing Link Workers on Health Outcomes and Costs for Adults in Primary Care and Community Settings: A Systematic Review. Dublin, Ireland: BMJ open. 10.1136/bmjopen-2022-062951 PMC9644316 36253037

