# Supplementary material for: Economic evidence related to the use of community assets in the management of frailty: a systematic review
Source: Front Public Health. 2026 Jul 9;14:1874340. doi: 10.3389/fpubh.2026.1874340 (PMC13394003; doi:10.3389/fpubh.2026.1874340)
Supplement: Supplementary file 2 [file Supplementary_file_1.DOCX]

# Search Strategy

This file present the search criteria that have been used in each database based on our keywords (Frailty, Community Assets, Economic Evaluation, Social Prescription, Physical Activity)

## PubMed, 5,605 results

(((((((((((("Frailty"[Mesh] OR "Frail Elderly"[Mesh] OR frail*[Mesh] OR "pre-frail"[Mesh] OR "pre frailty"[Mesh] OR "cognitive frailty"[Title/Abstract] ) OR (frail elderly)) OR (physical frailty)) OR (vulnerable older adults)) OR (age-related decline)) OR (geriatric)) OR (decline in physiological reserve)) OR (functional decline)) OR (frailty phenotype)) OR (frailty index)) OR (ageing-related vulnerability)) OR (sarcopenia)

AND

((((((((((((((((((((((((((((((((((((((((((("Social Prescribing"[Mesh] OR "Senior Center"[Mesh] OR "Community Assets"[Mesh] OR "Community Health Centers"[Mesh] OR "Community Resources"[Mesh]OR "Volunteers"[Mesh] OR social prescri*[Title/Abstract] OR senior center*[Title/Abstract] OR community center*[Title/Abstract] OR voluntary[Title/Abstract] OR volunteer*[Title/Abstract] OR "green prescri*"[Title/Abstract] OR "community-based"[Title/Abstract] OR "voluntary sector"[Title/Abstract] OR "third sector"[Title/Abstract] ) OR (wellbeing plan)) OR (Blue Referral)) OR (blue gym)) OR (swimming)) OR (rowing)) OR (surfing)) OR (Community Support Hubs)) OR (housing support)) OR (money advice)) OR (finding a job)) OR (lifestyle hubs)) OR (wellbeing hubs)) OR (Welfare Support Referral)) OR (Creative Referral)) OR (dance)) OR (photography)) OR (music)) OR (art)) OR (singing)) OR (museum referral)) OR (art referral)) OR (dance referral)) OR (art prescription)) OR (museum prescription)) OR (dance prescription)) OR (Exercise Referral)) OR (exercise prescription)) OR (Green Referral)) OR (green gym)) OR (green prescribing)) OR (gardening)) OR (hiking)) OR (woodland crafts)) OR (Nature-Based Interventions)) OR (Ecotherapy)) OR (Social Cafes)) OR (Reducing loneliness)) OR (Improving physical health)) OR (non-medical referral[MeSH Major Topic])) OR (community connection[MeSH Terms])) OR (community-based referral[MeSH Terms])) OR (social welfare)) OR (Welfare Support Referral)

AND

("Cost-Benefit Analysis"[Mesh] OR "Cost-Effectiveness Analysis"[Mesh] OR "Costs and Cost Analysis"[Mesh] OR "Economics"[Mesh] OR "Health Care Costs"[Mesh] OR "Health Economics"[Mesh] OR "Program Evaluation"[Mesh] OR "economic evaluation"[Title/Abstract] OR "cost*"[Title/Abstract] OR "econom*"[Title/Abstract] OR "cost-effectiveness"[Title/Abstract] OR "cost benefit"[Title/Abstract] OR "cost-benefit"[Title/Abstract] OR "cost-utility"[Title/Abstract] OR "cost analysis"[Title/Abstract] OR "economic burden"[Title/Abstract] OR "economic impact"[Title/Abstract] OR "cost evaluation"[Title/Abstract] OR "QALY"[Title/Abstract] OR "QALYs"[Title/Abstract] OR "quality-adjusted life year"[Title/Abstract] OR "quality-adjusted life years"[Title/Abstract] OR "SROI"[Title/Abstract] OR "social return on investment"[Title/Abstract] OR "CBA"[Title/Abstract])

-----------------

## Scoups: 1,339 results

(

TITLE-ABS-KEY(frail*) OR

TITLE-ABS-KEY("pre-frail") OR

TITLE-ABS-KEY("pre frailty") OR

TITLE-ABS-KEY("cognitive frailty") OR

TITLE-ABS-KEY("frail elderly") OR

TITLE-ABS-KEY("physical frailty") OR

TITLE-ABS-KEY("vulnerable older adults") OR

TITLE-ABS-KEY("age-related decline") OR

TITLE-ABS-KEY(geriatric) OR

TITLE-ABS-KEY("decline in physiological reserve") OR

TITLE-ABS-KEY("functional decline") OR

TITLE-ABS-KEY("frailty phenotype") OR

TITLE-ABS-KEY("frailty index") OR

TITLE-ABS-KEY("ageing-related vulnerability") OR

TITLE-ABS-KEY(sarcopenia)

)

AND

(

TITLE-ABS-KEY("social prescribing") OR

TITLE-ABS-KEY("senior center") OR

TITLE-ABS-KEY("community assets") OR

TITLE-ABS-KEY("community health center") OR

TITLE-ABS-KEY("community resources") OR

TITLE-ABS-KEY(volunteer*) OR

TITLE-ABS-KEY(voluntary) OR

TITLE-ABS-KEY("green prescribing") OR

TITLE-ABS-KEY("community-based") OR

TITLE-ABS-KEY("voluntary sector") OR

TITLE-ABS-KEY("third sector") OR

TITLE-ABS-KEY("wellbeing plan") OR

TITLE-ABS-KEY("blue referral") OR

TITLE-ABS-KEY("blue gym") OR

TITLE-ABS-KEY(swimming) OR

TITLE-ABS-KEY(rowing) OR

TITLE-ABS-KEY(surfing) OR

TITLE-ABS-KEY("community support hubs") OR

TITLE-ABS-KEY("housing support") OR

TITLE-ABS-KEY("money advice") OR

TITLE-ABS-KEY("finding a job") OR

TITLE-ABS-KEY("lifestyle hubs") OR

TITLE-ABS-KEY("wellbeing hubs") OR

TITLE-ABS-KEY("welfare support referral") OR

TITLE-ABS-KEY("creative referral") OR

TITLE-ABS-KEY(dance) OR

TITLE-ABS-KEY(photography) OR

TITLE-ABS-KEY(music) OR

TITLE-ABS-KEY(art) OR

TITLE-ABS-KEY(singing) OR

TITLE-ABS-KEY("museum referral") OR

TITLE-ABS-KEY("art referral") OR

TITLE-ABS-KEY("dance referral") OR

TITLE-ABS-KEY("art prescription") OR

TITLE-ABS-KEY("museum prescription") OR

TITLE-ABS-KEY("dance prescription") OR

TITLE-ABS-KEY("exercise referral") OR

TITLE-ABS-KEY("exercise prescription") OR

TITLE-ABS-KEY("green referral") OR

TITLE-ABS-KEY("green gym") OR

TITLE-ABS-KEY(gardening) OR

TITLE-ABS-KEY(hiking) OR

TITLE-ABS-KEY("woodland crafts") OR

TITLE-ABS-KEY("nature-based interventions") OR

TITLE-ABS-KEY(ecotherapy) OR

TITLE-ABS-KEY("social cafes") OR

TITLE-ABS-KEY("reducing loneliness") OR

TITLE-ABS-KEY("improving physical health") OR

TITLE-ABS-KEY("community connection") OR

TITLE-ABS-KEY("community-based referral") OR

TITLE-ABS-KEY("social welfare")

)

AND

(

TITLE-ABS-KEY("cost-benefit analysis") OR

TITLE-ABS-KEY("cost-effectiveness analysis") OR

TITLE-ABS-KEY("costs and cost analysis") OR

TITLE-ABS-KEY(economics) OR

TITLE-ABS-KEY("health care costs") OR

TITLE-ABS-KEY("health economics") OR

TITLE-ABS-KEY("program evaluation") OR

TITLE-ABS-KEY("economic evaluation") OR

TITLE-ABS-KEY(cost*) OR

TITLE-ABS-KEY(econom*) OR

TITLE-ABS-KEY("cost-effectiveness") OR

TITLE-ABS-KEY("cost benefit") OR

TITLE-ABS-KEY("cost-utility") OR

TITLE-ABS-KEY("cost analysis") OR

TITLE-ABS-KEY("economic burden") OR

TITLE-ABS-KEY("economic impact") OR

TITLE-ABS-KEY("cost evaluation") OR

TITLE-ABS-KEY(QALY) OR

TITLE-ABS-KEY(QALYs) OR

TITLE-ABS-KEY("quality-adjusted life year") OR

TITLE-ABS-KEY("quality-adjusted life years") OR

TITLE-ABS-KEY(SROI) OR

TITLE-ABS-KEY("social return on investment") OR

TITLE-ABS-KEY(CBA)

)

## Embase; 2,673 results

(('frailty'/exp OR 'frail elderly'/exp OR 'frail*' OR 'pre-frail' OR 'pre frailty'/exp OR 'cognitive frailty':ti,ab,kw OR 'frail elderly' OR 'physical frailty' OR 'vulnerable older adults' OR 'age-related decline' OR 'geriatric' OR 'decline in physiological reserve' OR 'functional decline' OR 'frailty phenotype' OR 'frailty index' OR 'ageing-related vulnerability' OR 'sarcopenia') AND ('social prescribing'/exp OR 'senior center'/exp OR 'community assets' OR 'health center'/exp OR 'community resource'/exp OR 'volunteer'/exp OR 'social prescri*':ti,ab,kw OR 'senior center*':ti,ab,kw OR 'community center*':ti,ab,kw OR 'voluntary':ti,ab,kw OR 'volunteer*':ti,ab,kw OR 'green prescri*':ti,ab,kw OR 'community-based':ti,ab,kw OR 'voluntary sector':ti,ab,kw OR 'third sector':ti,ab,kw OR 'wellbeing plan' OR 'blue referral' OR 'blue gym' OR 'swimming' OR 'rowing' OR 'surfing' OR 'community support hubs' OR 'housing support' OR 'money advice' OR 'finding a job' OR 'lifestyle hubs' OR 'wellbeing hubs' OR 'welfare support referral' OR 'creative referral' OR 'dance' OR 'photography' OR 'music' OR 'art' OR 'singing' OR 'museum referral' OR 'art referral' OR 'dance referral' OR 'art prescription' OR 'museum prescription' OR 'dance prescription' OR 'exercise referral' OR 'exercise prescription' OR 'green referral' OR 'green gym' OR 'green prescribing' OR 'gardening' OR 'hiking' OR 'woodland crafts' OR 'nature-based interventions' OR 'ecotherapy' OR 'social cafes' OR 'reducing loneliness' OR 'improving physical health' OR 'non-medical referral' OR 'community connection' OR 'community-based referral' OR 'social welfare') OR 'welfare support referral') AND ('cost benefit analysis'/exp OR 'cost effectiveness analysis'/exp OR 'cost'/exp OR 'economics'/exp OR 'health care cost'/exp OR 'health economics'/exp OR 'program evaluation'/exp OR 'economic evaluation':ti,ab,kw OR 'cost*':ti,ab,kw OR 'econom*':ti,ab,kw OR 'cost-effectiveness':ti,ab,kw OR 'cost benefit':ti,ab,kw OR 'cost-benefit':ti,ab,kw OR 'cost-utility':ti,ab,kw OR 'cost analysis':ti,ab,kw OR 'economic burden':ti,ab,kw OR 'economic impact':ti,ab,kw OR 'cost evaluation':ti,ab,kw OR 'qaly':ti,ab,kw OR 'qalys':ti,ab,kw OR 'quality-adjusted life year':ti,ab,kw OR 'quality-adjusted life years':ti,ab,kw OR 'sroi':ti,ab,kw OR 'social return on investment':ti,ab,kw OR 'cba':ti,ab,kw)

## Web of science; 546 results

(

TS=(frail*) OR

TS=("frail elderly") OR

TS=("pre-frail") OR

TS=("pre frailty") OR

TS=("cognitive frailty") OR

TS=("physical frailty") OR

TS=("vulnerable older adults") OR

TS=("age-related decline") OR

TS=(geriatric) OR

TS=("decline in physiological reserve") OR

TS=("functional decline") OR

TS=("frailty phenotype") OR

TS=("frailty index") OR

TS=("ageing-related vulnerability") OR

TS=(sarcopenia)

)

AND

(

TS=("social prescribing") OR

TS=("senior center*") OR

TS=("community center*") OR

TS=(voluntary) OR

TS=(volunteer*) OR

TS=("green prescri*") OR

TS=("community-based") OR

TS=("voluntary sector") OR

TS=("third sector") OR

TS=("wellbeing plan") OR

TS=("blue referral") OR

TS=("blue gym") OR

TS=(swimming) OR

TS=(rowing) OR

TS=(surfing) OR

TS=("community support hubs") OR

TS=("housing support") OR

TS=("money advice") OR

TS=("finding a job") OR

TS=("lifestyle hubs") OR

TS=("wellbeing hubs") OR

TS=("welfare support referral") OR

TS=("creative referral") OR

TS=(dance) OR

TS=(photography) OR

TS=(music) OR

TS=(art) OR

TS=(singing) OR

TS=("museum referral") OR

TS=("art referral") OR

TS=("dance referral") OR

TS=("art prescription") OR

TS=("museum prescription") OR

TS=("dance prescription") OR

TS=("exercise referral") OR

TS=("exercise prescription") OR

TS=("green referral") OR

TS=("green gym") OR

TS=("green prescribing") OR

TS=(gardening) OR

TS=(hiking) OR

TS=("woodland crafts") OR

TS=("nature-based interventions") OR

TS=(ecotherapy) OR

TS=("social cafes") OR

TS=("reducing loneliness") OR

TS=("improving physical health") OR

TS=("non-medical referral") OR

TS=("community connection") OR

TS=("community-based referral") OR

TS=("social welfare")

)

AND

(

TS=("cost benefit analysis") OR

TS=("cost effectiveness analysis") OR

TS=("costs and cost analysis") OR

TS=("economics") OR

TS=("health care costs") OR

TS=("health economics") OR

TS=("program evaluation") OR

TS=("economic evaluation") OR

TS=(cost*) OR

TS=(econom*) OR

TS=("cost effectiveness") OR

TS=("cost benefit") OR

TS=("cost-benefit") OR

TS=("cost-utility") OR

TS=("cost analysis") OR

TS=("economic burden") OR

TS=("economic impact") OR

TS=("cost evaluation") OR

TS=(QALY) OR

TS=(QALYs) OR

TS=("quality-adjusted life year") OR

TS=("quality-adjusted life years") OR

TS=(SROI) OR

TS=("social return on investment") OR

TS=(CBA)

)

## CINAHL Ultimate; 377 results

( MH "Frailty+" OR MH "Frail Elderly+" OR TI frail* OR AB frail* OR TI "pre-frail" OR AB "pre-frail" OR TI "pre frailty" OR AB "pre frailty" OR TI "cognitive frailty" OR AB "cognitive frailty" OR TI "physical frailty" OR AB "physical frailty" OR TI "vulnerable older adults" OR AB "vulnerable older adults" OR TI "age-related decline" OR AB "age-related decline" OR TI geriatric OR AB geriatric OR TI "decline in physiological reserve" OR AB "decline in physiological reserve" OR TI "functional decline" OR AB "functional decline" OR TI "frailty phenotype" OR AB "frailty phenotype" OR TI "frailty index" OR AB "frailty index" OR TI "ageing-related vulnerability" OR AB "ageing-related vulnerability" OR TI sarcopenia OR AB sarcopenia )

AND

( MH "Social Prescribing" OR MH "Senior Centers" OR MH "Community Health Services" OR MH "Community Resources" OR MH "Volunteers" OR TI "social prescri*" OR AB "social prescri*" OR TI "senior center*" OR AB "senior center*" OR TI "community center*" OR AB "community center*" OR TI voluntary OR AB voluntary OR TI volunteer* OR AB volunteer* OR TI "green prescri*" OR AB "green prescri*" OR TI "community-based" OR AB "community-based" OR TI "voluntary sector" OR AB "voluntary sector" OR TI "third sector" OR AB "third sector" OR TI wellbeing plan OR AB wellbeing plan OR TI "Blue Referral" OR AB "Blue Referral" OR TI "blue gym" OR AB "blue gym" OR TI swimming OR AB swimming OR TI rowing OR AB rowing OR TI surfing OR AB surfing OR TI "Community Support Hubs" OR AB "Community Support Hubs" OR TI "housing support" OR AB "housing support" OR TI "money advice" OR AB "money advice" OR TI "finding a job" OR AB "finding a job" OR TI "lifestyle hubs" OR AB "lifestyle hubs" OR TI "wellbeing hubs" OR AB "wellbeing hubs" OR TI "Welfare Support Referral" OR AB "Welfare Support Referral" OR TI "Creative Referral" OR AB "Creative Referral" OR TI dance OR AB dance OR TI photography OR AB photography OR TI music OR AB music OR TI art OR AB art OR TI singing OR AB singing OR TI "museum referral" OR AB "museum referral" OR TI "art referral" OR AB "art referral" OR TI "dance referral" OR AB "dance referral" OR TI "art prescription" OR AB "art prescription" OR TI "museum prescription" OR AB "museum prescription" OR TI "dance prescription" OR AB "dance prescription" OR TI "Exercise Referral" OR AB "Exercise Referral" OR TI "exercise prescription" OR AB "exercise prescription" OR TI "Green Referral" OR AB "Green Referral" OR TI "green gym" OR AB "green gym" OR TI "green prescribing" OR AB "green prescribing" OR TI gardening OR AB gardening OR TI hiking OR AB hiking OR TI "woodland crafts" OR AB "woodland crafts" OR TI "Nature-Based Interventions" OR AB "Nature-Based Interventions" OR TI Ecotherapy OR AB Ecotherapy OR TI "Social Cafes" OR AB "Social Cafes" OR TI "Reducing loneliness" OR AB "Reducing loneliness" OR TI "Improving physical health" OR AB "Improving physical health" OR TI "non-medical referral" OR AB "non-medical referral" OR TI "community connection" OR AB "community connection" OR TI "community-based referral" OR AB "community-based referral" OR TI "social welfare" OR AB "social welfare" )

AND

( MH "Cost Benefit Analysis" OR MH "Cost Effectiveness Analysis" OR MH "Costs and Cost Analysis" OR MH "Health Economics" OR MH "Health Care Costs" OR MH "Program Evaluation" OR TI cost* OR AB cost* OR TI econom* OR AB econom* OR TI "cost effectiveness" OR AB "cost effectiveness" OR TI "cost benefit" OR AB "cost benefit" OR TI "cost-benefit" OR AB "cost-benefit" OR TI "cost-utility" OR AB "cost-utility" OR TI "cost analysis" OR AB "cost analysis" OR TI "economic burden" OR AB "economic burden" OR TI "economic impact" OR AB "economic impact" OR TI "cost evaluation" OR AB "cost evaluation" OR TI QALY OR AB QALY OR TI QALYs OR AB QALYs OR TI "quality-adjusted life year" OR AB "quality-adjusted life year" OR TI "quality-adjusted life years" OR AB "quality-adjusted life years" OR TI SROI OR AB SROI OR TI "social return on investment" OR AB "social return on investment" OR TI CBA OR AB CBA )
